# Inhibition of interleukin-6 trans-signaling in the brain facilitates recovery from lipopolysaccharide-induced sickness behavior

**DOI:** 10.1186/1742-2094-8-54

**Published:** 2011-05-19

**Authors:** Michael D Burton, Nathan L Sparkman, Rodney W Johnson

**Affiliations:** 1 Laboratory of Integrative Immunology and Behavior, Animal Science Department, University of Illinois at Urbana-Champaign, Urbana, 7 Animal Sciences Lab 1207 W. Gregory Dr. Urbana IL 61801, USA

## Abstract

**Background:**

Interleukin (IL)-6 is produced in the brain during peripheral infection and plays an important but poorly understood role in sickness behavior. Therefore, this study investigated the capacity of soluble gp130 (sgp130), a natural inhibitor of the IL-6 trans-signaling pathway to regulate IL-6 production in microglia and neurons *in vitro *and its effects on lipopolysaccharide (LPS)-induced sickness behavior *in vivo*.

**Methods:**

A murine microglia (BV.2) and neuronal cell line (Neuro.2A) were used to study the effects of stimulating and inhibiting the IL-6 signaling pathway *in vitro. In vivo*, adult (3-6 mo) BALB/c mice received an intracerebroventricular (ICV) injection of sgp130 followed by an intraperitoneal (i.p.) injection of LPS, and sickness behavior and markers of neuroinflammation were measured.

**Results:**

Soluble gp130 attenuated IL-6- and LPS-stimulated IL-6 receptor (IL-6R) activation along with IL-6 protein release in both microglial (BV.2) and neuronal (Neuro.2A) cell types *in vitro*. Moreover, *in vivo *experiments showed that sgp130 facilitated recovery from LPS-induced sickness, and this sgp130-associated recovery was paralleled by reduced IL-6 receptor signaling, mRNA, and protein levels of IL-6 in the hippocampus.

**Conclusions:**

Taken together, the results show that sgp130 may exert an anti-inflammatory effect on microglia and neurons by inhibiting IL-6 binding. These data indicate that sgp130 inhibits the LPS-induced IL-6 trans-signal and show IL-6 and its receptor are involved in maintaining sickness behavior.

## Background

Peripheral infection stimulates production of pro-inflammatory cytokines including interleukin (IL)-1β, IL-6, and tumor necrosis factor-α (TNF-α). These cytokines use neural and humoral pathways to convey a message to the brain [1,2]. In the brain, the peripheral pro-inflammatory signal is mimicked by microglia, [3] and the resulting cytokines target neurons to elicit sickness- related behaviors that are typically adaptive [4]. However, excessive cytokine production by microglia is associated with prolonged sickness behavior [5-8], cognitive deficits [9-11], and affective disorders like anxiety and depression [12,13]. A recent study showed IL-6 knockout mice were refractory to LPS-induced increases of cytokines in the brain and cognitive deficits eluding to the potential permissive effects of IL-6 during LPS-induced sickness [14].

The IL-6 receptor is activated through two separate, but related pathways; classical- and trans-signaling. Classical IL-6 receptor activation is facilitated through the IL-6 ligand binding to its membrane-bound receptor. The receptor consists of two subunits: the IL-6 receptor-alpha chain (IL-6R), which binds IL-6, and the transmembrane signaling subunit, glycoprotein 130 (gp130), which is the intra-cellular signal transducer and is ubiquitously expressed. Both IL-6R and gp130 are cleaved immediately before the membrane spanning region by alternative splicing or shed by proteolytic enzymes to produce a soluble receptor located in extra-cellular matrix. It is important to note that the expression pattern of IL-6R is limited to few cells of the immune system and conservatively dispersed among other cell types, meaning classical signaling is highly conserved. In contrast, gp130 is ubiquitously expressed [15,16]. The basis of trans-signaling is soluble IL-6R (sIL-6R) binding to IL-6 in the extra cellular matrix to form a IL-6/sIL-6R complex, which has an increased binding affinity to membrane-bound gp130 subunits, resulting in the capability of IL-6 production in any cell type that expresses gp130 [17,18].

Upon binding through either the classical or trans-signal, gp130 dimerizes and autophosphorylates, resulting in the activation of Janus kinase-1 and 2 (Jak1 & Jak2). These tyrosine kinases phosphorylate the cytoplasmic region of gp130 creating recruitment sites for signal transducer and activation of transcription-3 (STAT3), a Src-homology-2 (SH2) domain-containing signaling molecule. Activated STAT3 forms a dimer, autophosphorylates, and translocates to the nucleus where it binds to enhancer elements of the IL-6 promoter region. Thus the main consequence of both classical or trans-signal IL-6 receptor action is to induce gene transcription and subsequent synthesis and secretion of IL-6, though trans-signaling allows this in many more cell types, due to the ubiquitous expression of gp130 [15]. sIL-6R and soluble gp130 (sgp130) have varying effects on circulating IL-6. Where sIL-6R acts as an agonist, sgp130 acts as a partial antagonist, or decoy receptor, by binding IL-6 or the IL-6/sIL-6R complex and prevents the binding of membrane-bound gp130 and further signal transduction [19].

The action of IL-6 is heavily dependent on the location of the receptors and the cell types exposed to the cytokine. For instance, IL-6 binding to IL-6R located on T-cells leads to the differentiation of stem line T-cells to helper T cells [20] whereas in the gastro-intestinal tract, IL-6 and its receptors on epithelial cells contribute to peripheral disorders such as colitis and Crohn's disease [21]. However, studies examining IL-6 receptor signaling or trans-signaling in the CNS are limited and we are aware of no studies examining the extent to which IL-6 receptor signaling affects neuroinflammation and infection-related changes in behavior.

Therefore the purpose of this study was to assess classical and trans-signaling in neurons and microglia and determine if inhibiting IL-6 receptor signaling in the brain is sufficient to inhibit sickness behavior caused by peripheral infection. The important results showed treatment with sgp130 attenuated LPS-induced receptor activation and production of IL-6 and enhanced recovery of sickness behavior. These findings suggest that inhibition of excessive production of IL-6 through its signaling pathways during infection may be helpful in preventing behavioral deficits.

## Methods

### BV.2 microglial and Neuro.2A neuronal cell culture

The murine microglia cell line, BV.2 (a gift from Linda Van Eldik, Northwestern University, Evanston, IL) and neuronal Neuro.2A cells (purchased from ATCC) have been used as a model to investigate the neuroimmune system [22,23]. Cells were maintained in 150-cm^2 ^tissue culture flasks (BD Falcon, Franklin Lakes, NJ) in DMEM (Bio-Whittaker, Cambrex, MD) supplemented with 10% FBS (Hyclone, Logan, UT), 200 mM glutamine, and 100 units/ml penicillin/streptomycin (Invitrogen, Carlsbad, CA) at 37°C in a humidified incubator under 5% CO_2_. Confluent cultures were passed by trypsinization. Cells were centrifuged (5 min at 27°C, 200 × *g*), and culture medium was removed. In all experiments, cells were re-suspended in DMEM supplemented with 10% FBS and seeded in six-well plates (BD Falcon, Franklin Lakes, NJ) at a population of 3 ×10^5 ^- 5 × 10^5 ^cells per well overnight at 37°C in a humidified incubator under 5% CO_2 _before treatments. Cells were treated with sterile saline containing 0.1% BSA (vehicle), sIL-6R, or sgp130 (R&D systems, Minneapolis, MN) for 1 h followed by treatment with recombinant IL-6 (R&D systems, Minneapolis, MN) or *Escherichia coli *LPS (serotype 0127:B8 Sigma, St. Louis, MO), for 20 min or 3 h, respectively.

### Flow cytometry

Flow cytometric analysis of microglial and neuronal cell surface markers was performed as described previously, with a few modifications [24]. In brief, Fc receptors on BV.2 microglia cells were blocked with anti-CD16/CD32 antibody (eBioscience, San Diego, CA) in a PBS-1% BSA/sodium azide solution, and incubated with anti-CD126 PE and anti-CD130 APC or anti-TLR-4 PE (eBiosciences, San Diego, CA), fluorescently labeled isotype antibodies for PE and APC (eBiosciences, San Diego, CA) were used for controls. Expression of surface receptors was determined using a Becton-Dickinson LSR II Flow Cytometer (Red Oaks, CA). Fifty thousand events were collected and flow data were analyzed using FCS Express software (De Novo Software, Los Angeles, CA).

### Animals and surgery

Adult (3-6 months) male BALB/c mice obtained from our in-house breeding colony were used in all experiments. Mice were housed in polypropylene cages and maintained at 21°C under a reverse-phase 12 h light-dark cycle (lights off at 07:00) with *ad libitum *access to water and rodent chow.

#### Surgery

Intracerebroventricular (ICV) cannulation was performed under aseptic conditions as described previously [25]. In brief, mice were deeply anesthetized with an intraperitoneal (i.p.) injection of ketamine and xylazine (100 and 10 mg/kg, respectively) and the surgical site was shaved and sterilized. They were positioned in a stereotaxic instrument (David Kopf Instruments, Tujunga, CA) so that the frontal and parietal bones of the skull were parallel to the surgical platform. An incision roughly 1.5 cm in length was made on the cranium to reveal the bregma and a 26-gauge stainless steel cannula (Plastics One, Roanoke, VA) was placed in the right lateral cerebral ventricle according to predetermined stereotaxic coordinates (lateral 1.6 mm and antero-posterior 1 mm to the bregma, and horizontal 2 mm from the dura mater). The cannula was secured using two adjacent stainless steel screws and cranioplastic cement (Plastics One, Roanoke, VA). A dummy cannula (Plastics One, Roanoke, VA) was inserted in the guide cannula to prevent occlusion and infection. Mice were injected subcutaneously with buprenorphine (0.05 mg/kg) following surgery and then again 8-12 h later to aid with any post-operative discomfort. Mice were provided a minimum of seven days to recover from any discomfort or weight loss before any treatment or behavioral test. Accurate placement of the cannula was confirmed by allowing 2 μl of sterile saline to flow via gravity into the lateral ventricle. If cannula placement could not be confirmed, the animal was excluded from the study. All procedures were in accordance with the National Institutes of Health Guidelines for the Care and Use of Laboratory Animals and were approved by the University of Illinois Institutional Animal Care and Use Committee.

### Animal studies

Mice were handled 1-2 min each day for seven days before experimentation to acclimate them to routine handling. On test day, animals were injected ICV with sterile saline containing 0.1% BSA (vehicle) or 100 ng sgp130 dissolved in 2 μl vehicle. At the same time as the ICV injection, mice were injected i.p. with sterile saline or LPS (0.33 mg/kg BW or 10 μg, serotype 0127:B8, Sigma, St. Louis, Mo.). The LPS dosage was selected because it elicits a proinflammatory cytokine response in the brain, which results in mild transient sickness behavior in adult mice [26]. Tests were conducted during the dark phase (between 07:00 and 19:00) of the light/dark cycle under infrared lighting to aid video recording. Baseline behavior was taken just before treatment administration (0 h) and 4, 8, and 24 h afterwards.

To measure changes in cytokines and signaling molecules, mice not exposed to the behavior paradigms were injected ICV with vehicle or sgp130 (100 ng) and i.p. with sterile saline or LPS (10 μg) and killed 8 h later by CO_2 _asphyxiation. Blood samples were collected via cardiac puncture into EDTA-coated syringes to obtain plasma, and the brain was rapidly removed and dissected to obtain hippocampal tissue. Plasma and hippocampal tissue were snap-frozen in liquid nitrogen and stored at -80°C until later analysis.

### Behavioral tests

#### Social exploratory behavior

To assess motivation to engage in social exploration, a novel male juvenile conspecific (20-30 days old) from our in-house colony was introduced into the test subject's home cage for a 7 min period. Mice were video recorded, and the duration engaged in social investigation was determined from the video records by a trained observer who was blind to experimental treatments. Social behavior was determined as the amount of time that the experimental animal spent investigating (e.g. trailing, anogenital sniffing) the juvenile. Baseline social behavior was determined for all experimental treatments at the 0 h, for a 7 min period. Statistical analysis revealed there were no significant differences between treatment groups at baseline. The results are expressed as percent depression in time engaged in social behavior compared to respective baseline measures.

### Western immunoblotting

To assess IL-6 receptor signaling in CNS cells *in vitro*, BV.2 and Neuro.2A cells were harvested and *in vivo*, mouse hippocampal tissue was unthawed, and lysed in ice cold lysis buffer containing: 100 mM HEPES (7.5 pH), 150 mM NaCl, 1% Nonidet P-40 (U.S. Biological, Swampscott, MA), 2 mM EGTA, 2 mM Sodium Orthovanadate, Protease Inhibitor cocktail (100 mM EDTA, 1 μg/mL AEBSF, Bestatin, Pepstatin A, Leupeptin, Aprotinin, and E-64), and 1 mM PMSF and centrifuged at 11000 × g for 10 min at 4°C to remove all cellular debris. Protein concentration was determined using the BCA Protein Assay according to the manufacturer's protocol (Bio-Rad, Hercules, CA). Lysate concentration was then normalized and denatured in SDS/PAGE buffer at 95°C and stored at -20°C until use. All lysates were electrophoresed and separated on a 7.5% SDS-PAGE gel, and transferred onto nitrocellulose membranes (GE Healthcare, Minneapolis, MN). The membranes were blocked with 5% non-fat milk and incubated with anti-phosphorylated STAT3 (tyr-705) antibody (Cell Signaling, Danvers, MA) overnight at 4°C. After incubation with an HRP-conjugated secondary antibody, the protein bands were detected with a chemiluminescenct substrate (Cell Signaling, Danvers, MA) and Bio-Max film (Eastman Kodak Company, Rochester, NY). For detection of total STAT3 protein, the membranes were stripped with stripping buffer (2% SDS, 6.25 mM Tris. HCL (6.8 pH), 0.70% β-ME) followed by overnight incubation with anti-STAT3 antibody (Cell Signaling, Danvers, MA) at 4°C. Immunoblot results were quantified using ImageJ 1.41 software (NIH).

### Cytokine detection in cell supernatant, hippocampus, and plasma

Hippocampal tissue was lysed in ice cold lysis buffer and protein concentrations were determined using the BCA protein assay according to manufacturer's protocol. For hippocampal tissue, the antibodies and standards for the IL-6 ELISA were used according to the description by the manufacturer (eBiosciences San Diego, CA). Cell supernatants and plasma samples were assayed for IL-1β, TNF-α, IL-6, and the anti-inflammatory cytokine IL-10, using multiplexed bead-based immunoassay kits combined with a Cytokine Reagent kit as described by the manufacturer (Bio-Rad, Hercules, CA).

### Cytokine mRNA measurement by quantitative real-time PCR

Total RNA from hippocampus was isolated using the Tri Reagent protocol (Sigma, St. Louis, MO.) A QuantiTect Reverse Transcription Kit (Qiagen, Valencia, CA) was used for cDNA synthesis with integrated removal of genomic DNA contamination according to the manufacturer's protocol. Quantitative real time PCR was performed using the Applied Biosystems (Foster, CA) Assay-on Demand Gene Expression protocol as previously described [27]. In brief, cDNA was amplified by PCR where a target cDNA (IL-6, Mm00446190_m1; IL-1β, Mm00434228_m1; TNF-α, Mm00443258_m1; IL-10, Mm00439616_m1, IL-6R, 00439653_m1; and gp130, mM00439665_m1) and a reference cDNA (glucose-3 phosphate dehydrogenase, Mm99999915_g1) were amplified simultaneously using an oligonucleotide probe with a 5' fluorescent reporter dye (6-FAM) and a 3' quencher dye (NFQ). PCR reactions were performed in triplicate under the following conditions: 50°C for 2 min, 95°C for 10 min, followed by 40 cycles of 95°C for 15 sec and 60°C for 1 min. Fluorescence was determined on an ABI PRISM 7900HT-sequence detection system (Perkin Elmer, Forest City, CA). Data were analyzed using the comparative threshold cycle (Ct) method, and results are expressed as fold difference.

### Statistical analysis

All data were analyzed using Statview and Statistical Analysis System software (SAS Inst., Cary, NC). Behavioral data were subjected to a three-way ANOVA (sgp130 × LPS × Time) using repeated measures in which Time (0, 4, 8, and 24 h) was a within subjects measure, and sgp130 and LPS were between subjects measures. Cytokine mRNA and protein levels were analyzed using a two-way ANOVA (sgp130 × LPS). Phosphorylation of STAT3 levels were analyzed using a two-way ANOVA (sIL-6R or sgp130 × LPS). Post hoc Student's *t *test of least square means was used to determine if treatment means were significantly different from one another (*P <*0.05). All data are presented as mean ± SEM.

## Results

### IL-6 and LPS induce STAT3 phosphorylation in microglia and neurons

**Figure 1:**
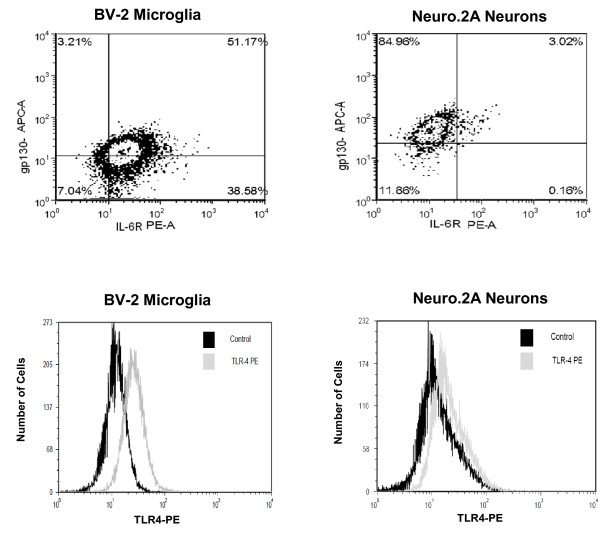
**Differential expression of IL-6R, gp130, and TLR-4 on microglia and neurons**. A) Two-color dot plot; cells were incubated with anti-CD126 PE and anti-CD130 APC and expression of the cell surface markers were assessed on microglia and neurons. B) Single-parameter histograms; cells were incubated with anti-TLR-4 and compared with isotype controls.

**Figure 2:**
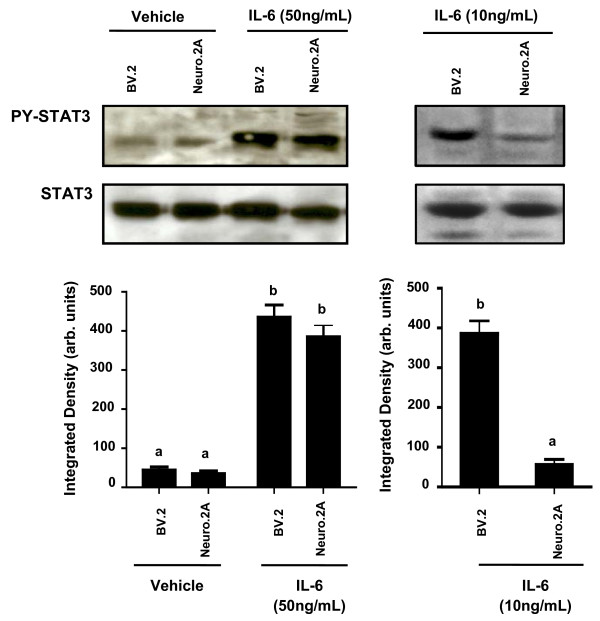
**IL-6 and STAT3 expression in BV.2 microglial and Neuro.2A neuronal cells**. IL-6 receptor activation in BV.2 and Neuro.2A cells was verified by STAT3 phosphorylation 20 min after treatment with 10 and 50 ng/mL of IL-6. Results are an average of 5 independent experiments. Means with different letters are significantly different from one another (P < 0.05).

To verify the presence of the subunits involved in IL-6 and LPS signaling, the cell surface expression of IL-6R, gp130, and TLR-4 on BV.2 and Neuro.2A cells was examined. Figure 1A shows more than 50% of the microglial BV.2 cells expressed gp130 while nearly 90% expressed IL-6R; approximately 50% of the BV.2 cells expressed both IL-6R and gp130. In contrast, about 90% of the Neuro.2A cells expressed gp130, 3% expressed IL-6R, and 3% co-expressed IL-6R and gp130. Approximately 80% of the BV.2 cells expressed TLR-4 compared to 30% of the Neuro.2A cells (Figure 1B). Although IL-6 can activate multiple transcription factors (e.g., STAT3, AP-1, CREB), in CNS cells activation of the IL-6 receptor upregulates STAT3 phosphorylation [15,28,29]. Thus, the capacity of IL-6 to induce the phosphorylation of STAT3 in BV.2 and Neuro.2A cell cultures was examined. Figure 2 shows that IL-6 at a higher concentration (50 ng/mL) increased phosphorylated STAT3 similarly in microglia [F (1,48) = 12.515, p < 0.001] and neurons [F (1,50) = 11.115, p < 0.01]. However, at a lower concentration (10 ng/mL), IL-6 only increased STAT3 phosphorylation in microglia [F (1, 14) = 37.384, P <.001], which is consistent with the greater proportion of these cells that expressed IL-6R. These findings suggest that classic and trans-signaling can occur on both neurons and microglia, although neurons may be more readily regulated through the mechanism of trans-signaling since gp130 is highly expressed on this cell type.

### IL-6 trans-signaling in microglia and neurons

**Figure 3:**
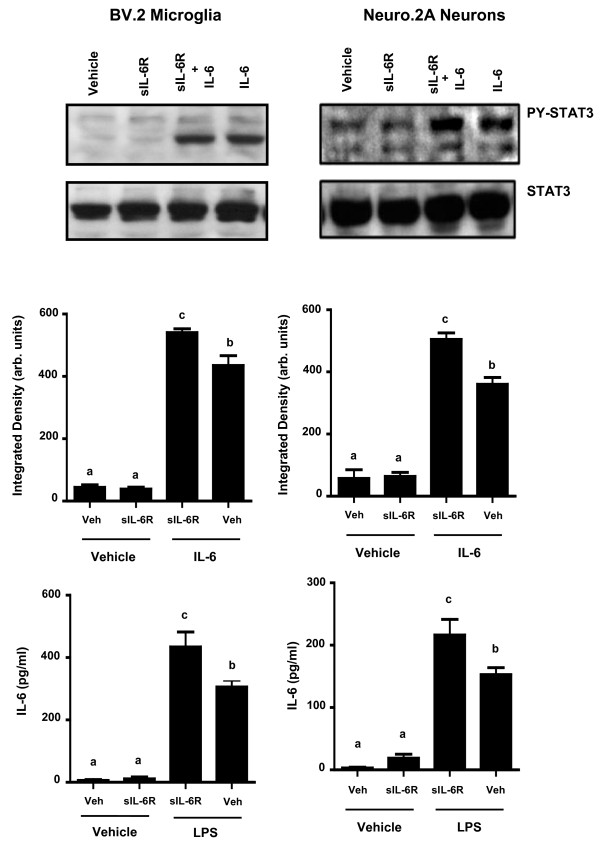
**IL-6 trans-signaling in BV.2 microglia and Neuro.2A cells**. BV.2 and Neuro2A cells were pre-treated for 1 h with 25 ng/mL sIL-6R and A) IL-6-induced STAT3 phosphorylation and B) LPS-induced IL-6 protein secretion were measured at 20 min and 3 h, respectively. Results are an average of 5 independent experiments. Means with different letters are significantly different from one another (P < 0.05).

Previous studies have shown that gp130 is expressed constitutively on all cell types [30,31] and this expression facilitates trans-signaling in the presence of IL-6 and sIL-6R [17,18,32]. Figure 3A shows that pretreatment of microglia and neurons with sIL-6R increased IL-6-induced STAT3 phosphorylation [F (2, 55) = 4.963, p < 0.01] and [F (2,53) = 7.642, p < 0.001], respectively. Consistent with the increase in STAT3 phosphorylation, a sIL-6R × LPS interaction was evident whereby sIL-6R upregulated LPS-induced IL-6 production in microglia [F (2,55) = 3.419, p < 0.05], and neurons [F (2,53) = 3.619, p < 0.04]. Although not statistically significant, there was some constitutive STAT3 phosphorylation and IL-6 expression in samples pretreated with sIL-6R (Figure 3A and 3B).

### sgp130 attenuated IL-6R activation in microglia and neurons

**Figure 4:**
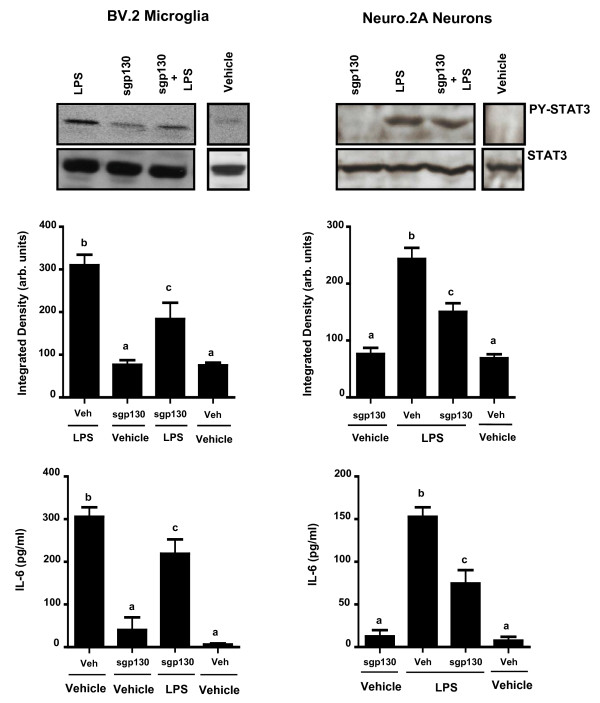
**sgp130 attenuation of LPS-induced STAT3 phosphorylation in Neuro.2A and BV.2 cells**. BV.2 microglia and Neuro.2A neuronal cells were pre-treated for 1 h with 100 ng sgp130 and LPS-induced A) STAT3 phosphorylation and B) IL-6 protein secretion were measured. Results are an average of 5 independent experiments. Means with different letters are significantly different from each other (P < 0.05).

We next investigated the ability of sgp130 to alter phosphorylation of STAT3 and expression of IL-6. A sgp130 × LPS interaction revealed that pretreatment of BV.2 microglial and Neuro.2A neuronal cells with sgp130 decreased LPS-induced activation of STAT3 [F (2,59) = 3.736, p < 0.03] and [F (2,60) = 4.385, p < 0.02], respectively and inhibited LPS-induced IL-6 production in both BV.2 [F (2,59) = 3.253, p < 0.05] and Neuro.2A cells [F (2,60) = 3.680, p < 0.04] (Figure 4A and 4B, respectively). These data demonstrate that sgp130 inhibits LPS-induced IL-6 production in microglia and neurons. The LPS-induced secretion of IL-1β, TNF-α, and IL-10 was not affected by sgp130 (data not shown).

### sgp130 inhibits LPS-induced sickness behavior

**Figure 5:**
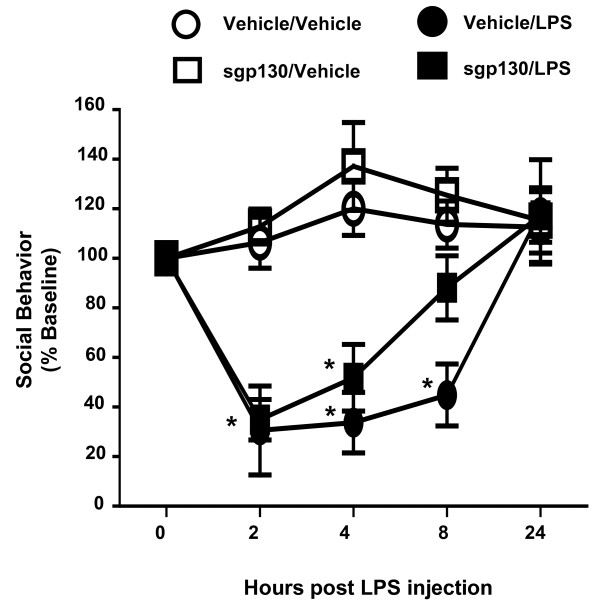
**sgp130 facilitates recovery from LPS-induced sickness behavior**. Mice were injected ICV with vehicle or 100 ng sgp130 and i.p. with sterile saline or LPS. Social exploratory baseline behavior was measured before LPS injection and at 2, 4, 6, 8, and 24 h post-injection. Bars represent the mean ± SEM (n = 11-12) Means with * are statistically different (P < 0.05) from saline controls.

Brain microglia and neurons produce inflammatory cytokines, including IL-6, that induce sickness behavior. Given the *in vitro *results, we investigated the effect of centrally administered sgp130 on LPS-induced sickness behavior. Social exploratory behavior was used to measure sickness. Three-way ANOVA of social behavior revealed a significant LPS × time × sgp130 interaction [F (3,48) = 6.280, P < 0.01]. As expected, LPS treatment decreased social exploratory behavior in a time-dependent manner [F (3,48) = 15.896, p < 0.001]. LPS induced transient sickness, as the behavior of mice given LPS returned to baseline by 24 h post-injection (Figure 5). However, behavior of mice treated ICV with sgp130 prior to LPS returned to normal sooner. That sgp130 did not inhibit LPS-induced sickness behavior at 2 or 4 h after LPS treatment but did later, suggests the IL-6 trans-signaling pathway is important for maintaining sickness behavior but not for its induction.

### sgp130 attenuated STAT3 phosphorylation and IL-6 gene expression and protein in the brain

**Figure 6:**
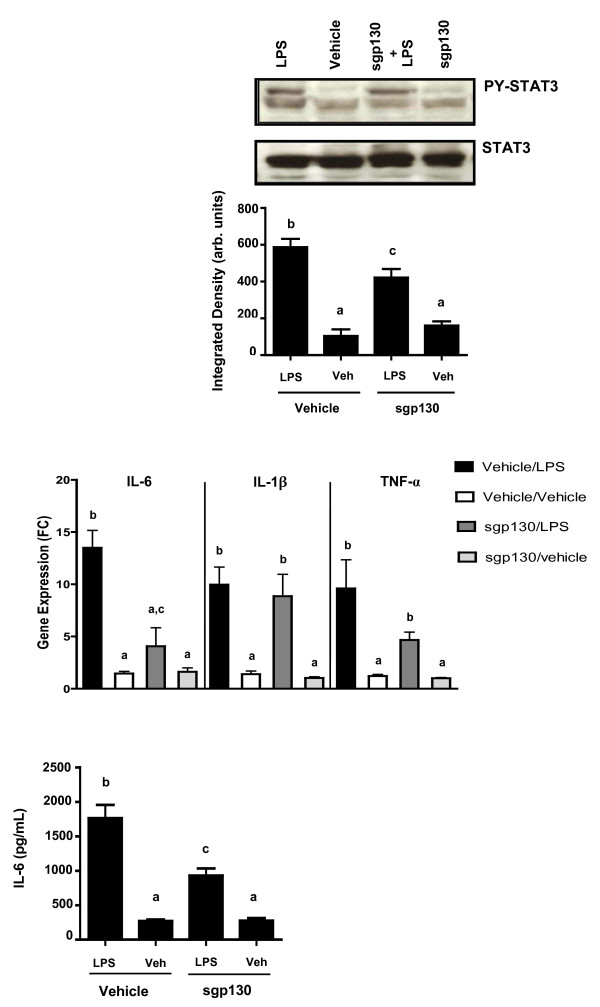
**sgp130 reduces IL-6 receptor activation, gene expression, and protein secretion *in vivo***. Hippocampal tissue was collected 8 h after ICV sgp130 and i.p. LPS and assayed for A) STAT3 phosphorylation, B) mRNA levels of IL-6¸IL-1β, and TNF-α, C) and IL-6 protein. sgp130 reduced LPS-induced STAT3 phosphorylation, mRNA levels of IL-6, but not IL-1β or TNF-α, and IL-6 protein in the hippocampus. Bars represent the mean ± SEM (n = 8-9) Means with different letters are statistically different from each other (P < 0.05).

Because sgp130 inhibited LPS-induced sickness behavior 8 h post-injection, hippocampal tissue and plasma was collected from a separate group of sgp130- and LPS-treated mice at the 8 h time point to assess STAT3 phosphorylation and IL-6 expression. Similar to the *in vitro *results, i.p. LPS upregulated STAT3 phospho-protein in the hippocampus [F (1, 19) = 369.003, p < 0.0001]. There was a sgp130 × LPS interaction [F (1,19) = 22.293, P < 0.001] whereby STAT3 phosphorylation was blunted when mice were given ICV sgp130 (Figure 6A). There was also a significant sgp130 × LPS interaction [F (1,19) = 5.108, P < 0.02] whereby sgp130 decreased the amount of LPS-induced IL-6 mRNA in the hippocampus, although it did not significantly affect IL-1β or TNF-α mRNA (Figure 6B). To determine if the effect of sgp130 was also apparent at the protein level, LPS-induced IL-6 protein was measured. As expected, LPS alone increased IL-6 protein in the hippocampus; however, a sgp130 × LPS interaction [F (1,21) = 42.921, p < 0.0001] indicated that co-administration of sgp130 inhibited the LPS-induced increase in IL-6 (Figure 6C). Taken together, these results show that sgp130-related changes in LPS-induced social behavior are paralleled by sgp130-associated changes in the brain.

To assess the effect of ICV sgp130 on the peripheral cytokine response to i.p. LPS, plasma was assayed for IL-1β, IL-6, IL-10, and TNF-α. Plasma levels of all four cytokines was increased after LPS treatment and this was not affected by sgp130 (Table 1), suggesting ICV sgp130 acts locally in the brain.

**Table 1:** Plasma cytokines 8 h after sgp130 and LPS

	Vehicle (ICV)		sgp130 (100 ng) (ICV)		p < 0.05 interaction		
	**Saline (i.p.)**	**LPS (10 μg) (i.p.)**	**Saline (i.p.)**	**LPS (10 μg) (i.p.)**	**sgp130**	**LPS**	**sgp130 × LPS**

IL-1β	206.82 ± 86.19	1113.12 ± 24.48	191.10 ± 85.12	1543 ± 695.96	0.537	0.009*	0.513
IL-6	142.87 ± 117.79	2873.03 ± 994.50	96.50 ± 8.41	2682.06 ± 669.86	0.820	0.001*	0.955
TNF-α	325.89 ± 54.60	987.55 ± 104.77	322.95 ± 73.31	947.93 ± 119.32	0.829	< 0.0001*	0.852
IL-10	56.39 ± 23.01	407.34 ± 92.56	46.72 ± 11.47	447.22 ± 174.13	0.764	0.002*	0.719

## Discussion

Bi-directional communication between the periphery and the brain is important for the appropriate response to an immune stimulus [33]. During peripheral infection, pro-inflammatory cytokines are produced in the brain and play a role in adaptive sickness behavior. However, an excessive cytokine response in the brain is associated with prolonged sickness behavior [5-8], cognitive deficits [9-11], and increased anxiety [12,13]; and the specific role of IL-6 has not been extensively studied. We investigated the capacity of sgp130 to block IL-6 production in microglia and neurons *in vitro *and explored the effects of sgp130 on LPS-induced sickness behavior *in vivo. In vitro*, sgp130 attenuated LPS-stimulated IL-6R activation along with IL-6 protein release in both microglial (BV.2) and neuronal (Neuro.2A) cell types. Moreover, *in vivo *experiments showed that ICV sgp130 facilitated recovery from LPS-induced sickness, and this sgp130-associated recovery was paralleled by reduced IL-6 mRNA and protein levels in the hippocampus.

The present study demonstrates that murine microglia and neurons have the potential to produce IL-6 through both a classic and trans-signaling pathway. In two-color flow cytometry experiments, we showed that BV.2 and Neuro.2A cells expressed both gp130 and IL-6R on their cell surface, though expression differed in each cell type. The findings indicate that classic and trans-signaling are important on both neurons and microglia, though neurons may be more readily regulated through the mechanism of trans-signaling. Previous studies report that the presence of the sIL-6R elicits an exaggerated production of IL-6 protein [32,34,35]. Consistent with these reports, we found that pretreatment of sIL-6R led to an IL-6- and LPS-induced increase of IL-6 protein in microglia and neurons. This response is presumably elicited by the ligand and soluble receptor forming a sIL-6R/IL-6 complex. This complex has the ability to bind to the gp130 transmembrane receptor signal transducer and activate intracellular signals that produce IL-6 in any cell type via this trans-signaling mechanism.

LPS binds TLR-4, which we confirmed was present on both microglia and neurons. Upon binding, LPS induces upregulation of the NF-κB transcription factor that binds promoter regions to stimulate the production of IL-6 along with a milieu of other cytokines (e.g. IL-1β, TNF-α, and IL-10) [36]. Soluble gp130 inhibits IL-6 trans-signaling but also regulates IL-6 related cytokines oncostatin M (OSM) and leukemia inhibitory factor (LIF). However, sgp130 has a much lower affinity for OSM and LIF than for the IL-6/sIL-6R complex [19] and would not be expected to affect either cytokine at the dose used here [19,37]. Therefore, using sgp130 allowed us to investigate the effects of IL-6 after LPS treatment, while leaving all other cytokines unaffected.

Successful activation of the IL-6R is noted by the dimerization of gp130, resulting in an intracellular cascade that forms recruitment sites for STAT3 in the cytoplasmic region. STAT3 homodimerizes, autophosphorylates, then translocates to the nucleus and binds to enhancer elements of IL-6 to induce gene transcription. Here, STAT3 was upregulated in response to both IL-6 and LPS in BV.2 and Neuro.2A cells and pretreatment with sIL-6R led to an increased IL-6- and LPS-induced STAT3 phosphorylation. However, when pretreated with sgp130, IL-6 and LPS-stimulated BV.2 and Neuro.2A cells displayed a decrease in STAT3 phosphorylation. These data agree with other studies using sgp130 to inhibit IL-6 signaling in peripheral models of inflammation such as arthritis, peritonitis, and colitis [38-40]. To our knowledge, this is the first study to report that pretreatment with sgp130 attenuated LPS-induced IL-6 protein secretion in CNS-derived cells.

LPS activation of the peripheral innate immune system stimulates a robust secretion of inflammatory cytokines through the NF-κB pathway and these cytokines are relayed to the CNS via vagal nerve afferents, and humoral and diffusive pathways [1,2]. Once in the brain this inflammatory signal is mimicked by innate immune cells [3,41,42] and targets neurons which elicit a sickness behavior response that includes general malaise, decreased activity, decreased social interaction, decreased food and water intake (weight loss), and sleep dysregulation [4,33,43]. We therefore investigated the effects of ICV sgp130 *in vivo *and hypothesized that, given the role of IL-6 in neuroinflammatory responses; it would attenuate LPS-induced sickness behavior and IL-6 production. Here we show that sgp130 was effective in facilitating the recovery from LPS-induced social exploratory behavior as early as 8 h in mice. In addition to facilitating recovery from LPS, sgp130 attenuated receptor activation, gene expression, and production of IL-6 in adult mice 8 h after LPS injection. Consistent with previous studies [24,26,44,45], a reduction in brain cytokines did not prevent the initial induction of LPS-induced sickness behavior seen at 2-4 h post-injection, but rather facilitated the recovery from sickness starting at the 8 h time point [32,34,35]. In this model, the inability of sgp130 to block the onset of sickness behavior can be attributed to the fact that LPS induces multiple proinflammatory cytokines that have redundant properties and inhibition of a single cytokine is not sufficient to block the initial sickness. It is noteworthy that a study showed that LPS-induced sickness behavior was blocked only if IL-1β, IL-6, and TNF-α were antagonized simultaneously [46].

This facilitation in recovery from LPS-induced sickness has been observed in various nutritional and pharmacological interventions [5,47-49] and may be of particular importance when considering conditions where an exaggerated response is elicited during a primed inflammatory state, such as in overexpressing transgenic animals [9,11], prion disease [6], and aging [7,8,10]. We have previously demonstrated that aged animals display an exaggerated neuroinflammatory and sickness behavior response after activation of the peripheral immune system [7] and it appears that primed microglia are responsible for this exacerbated phenotype [13,44,50]. We and others have shown interventions that are anti-inflammatory are able to ameliorate the exaggerated cytokine response in the brain as well as the mal-adaptive behavioral response that results from peripheral infection [5,24,47-49,51]. Based on the data obtained from this study, it is possible that sgp130 will abrogate the prolonged LPS-induced alterations in sickness behavior, cognition, as well as exaggerated IL-6 levels exhibited in aged mice.

## Conclusion

Studies have highlighted the potential therapeutic role of sgp130 in treating inflammation; it has been shown to suppress the severity of experimentally-induced arthritis, modulate leukocyte trafficking, and mitigate the effects of colitis and colon cancer [18,40,52-54]. The current study is the first to extend the body of literature and show the effectiveness of sgp130 in inhibiting IL-6 signaling in cells of the CNS and in brains of animals. The present results suggest that the use of sgp130 as an inhibitor of the IL-6 pathway in an array of inflammatory conditions, from arthritis to neuroinflammatory disorders, may mitigate IL-6 expression and have a beneficial effect on behavioral responses.

## Competing interests

The authors declare that they have no competing interests.

## List of abbreviations

(AP-1): Activator Protein-1; (AEBSF): 4-(2-Aminoethyl) benzenesulfonyl fluoride hydrochloride; (APC): Allophycocyanin; (ANOVA): Analysis of variance; (BCA): Bicinchoninic acid; (BSA): Bovine serum albumin; (CNS): Central nervous system; (CREB): cAMP response element-binding; (DMEM): Dulbecco's modified eagle's medium; (EDTA): Ethylene diamine tetraacetic acid; (EGTA): Ethylene glycol tetraacetic acid; (ELISA): Enzyme-linked immmunosorbent assay; (FITC): Fluorescein isothiocyanate; (FBS): Fetal bovine serum; (HBSS): Hank's balanced salt solution; (HEPES): 4-(2-hydroxyethyl)-1-piperazineethanesulfonic acid; (HRP): Horseradish peroxidise; (ICV): Intracerebroventricular; (i.p.): Intraperitoneal; (NFκB): Nuclear factor kappa B; (PE): R-Phycoerthrin; (PMSF): Phenylmethylsulfonyl fluoride; (SAS): Statistical Analysis Systems; (SEM): Standard error of the mean; (SDS): Sodium dodecyl sulphate; (TLR4): Toll-like receptor-4; and (tyr): Tyrosine.

## Authors' contributions

MDB was involved in research experimentation, completion of statistical analysis, and writing of the manuscript. NLS assisted with experimentation and data analysis. RWJ directed all aspects of this research project including experimental design, research experimentation, completion of statistical analysis, and writing of the manuscript. All authors have read and approved the final version of the manuscript.
